# Synaptotagmin-1 membrane binding is driven by the C2B domain and assisted cooperatively by the C2A domain

**DOI:** 10.1038/s41598-020-74923-y

**Published:** 2020-10-22

**Authors:** Clémence Gruget, Oscar Bello, Jeff Coleman, Shyam S. Krishnakumar, Eric Perez, James E. Rothman, Frederic Pincet, Stephen H. Donaldson

**Affiliations:** 1 Laboratoire de Physique de L’Ecole Normale Supérieure, ENS, Université PSL, CNRS, Sorbonne Université, Université de Paris, 75005 Paris, France; 2 Department of Cell Biology, Yale University School of Medicine, New Haven, CT USA; 3 Department of Clinical and Experimental Epilepsy, Institute of Neurology, University College London, London, UK; 4 Present Address: W.L. Gore & Associates, Inc, 3750 W. Kiltie Lane, Flagstaff, AZ USA

**Keywords:** Exocytosis, Molecular conformation

## Abstract

Synaptotagmin interaction with anionic lipid (phosphatidylserine/phosphatidylinositol) containing membranes, both in the absence and presence of calcium ions (Ca^2+^), is critical to its central role in orchestrating neurotransmitter release. The molecular surfaces involved, namely the conserved polylysine motif in the C2B domain and Ca^2+^-binding aliphatic loops on both C2A and C2B domains, are known. Here we use surface force apparatus combined with systematic mutational analysis of the functional surfaces to directly measure Syt1-membrane interaction and fully map the site-binding energetics of Syt1 both in the absence and presence of Ca^2+^. By correlating energetics data with the molecular rearrangements measured during confinement, we find that both C2 domains cooperate in membrane binding, with the C2B domain functioning as the main energetic driver, and the C2A domain acting as a facilitator.

## Introduction

Coherent cognitive activity depends on the speed and synchronicity of synaptic transmission. This requires synaptic vesicles filled with neurotransmitters to fuse with the plasma membrane of the neuron in order to release their contents in a sub-millisecond timescale in response to the influx of Ca^2+^ ions following an action potential. Several proteins are essential to orchestrate this process. Synaptotamgmin-1 (Syt1), a synaptic vesicle associated protein, has been identified as the principal calcium-sensor that triggers the full assembly of the soluble N-ethylmaleimide-sensitive factor activating protein receptor (SNARE) proteins upon Ca^2+^ binding, leading to rapid and synchronized membrane fusion^1–4^.

**Figure 1:**
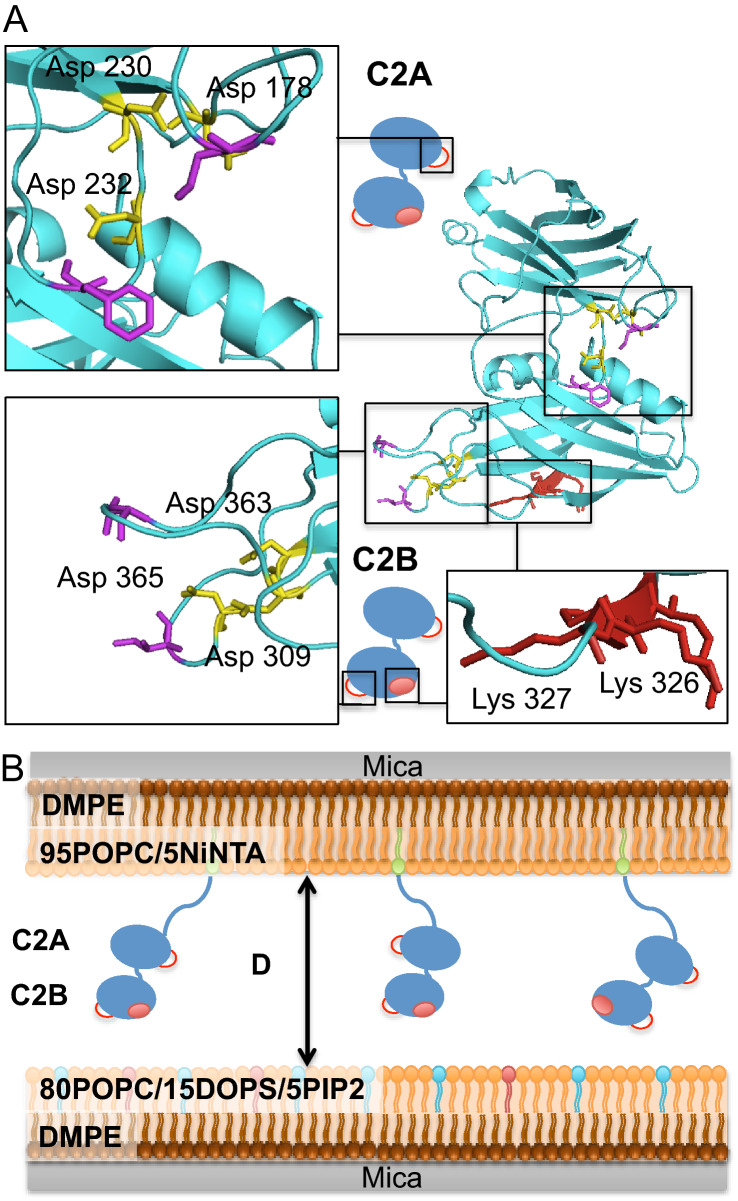
(**A**) Crystallographic structure of the Synaptotagmin-1 C2AB domain (code PDB 2R83). Insights show the mutations introduced in functional sites of Syt1 in this study. Yellow: calcium binding sites of C2A and C2B. Three aspartates were mutated to alanines in each site (D178/230/232A in C2A and D309/363/365A in C2B) to disrupt the coordination of calcium ions. Purple: calcium loops. Red: calcium-independent lipid-binding site consisting of four lysine residues (K324, K325, K326, K327). Two lysines were mutated to alanines (K326/327A or KAKA mutant). (**B**) A schematic of the SFA experiment. Syt-1 coated membrane (top) interacts with an anionic membrane (bottom) composed of 80% POPC, 15% DOPS and 5% PIP2. Syt1 calcium binding sites of C2A and C2B are indicated by the red loops and the C2B polylysine site is indicated by the red dot.

Syt1 is associated with the synaptic vesicle via its N-terminal transmembrane helix. A 61-residue linker connects the transmembrane domain and two tandem C2 domains, called C2A and C2B, which are separated by a 9-residue flexible linker domain (Fig. 1). The C2 domains confer distinct Ca^2+^-independent and Ca^2+^-dependent properties, as expected from Syt1′s role as a calcium-sensor. They are composed of a stable eight-stranded β-sandwich with flexible loops emerging from the top and the bottom. Two distinct loops at the top of each C2 domains, termed loop 1 and loop 3, form a negatively charged pocket that can coordinate Ca^2+^ ions. Syt1 C2A domain ligates three Ca^2+^ ions via five aspartate and one serine residues (D172, D178, D230, D232, D238, S235) while the C2B binds two Ca^2+^ ions via five aspartate residues (D303, D309, D363, D365, D371)^5–12^ (Fig. 1A). In both C2A and C2B, coordination of Ca^2+^ ions is incomplete. This allows for a Ca^2+^-dependent binding of Syt1 to a ternary component that can complete the Ca^2+^ coordination sphere, such as phospholipids. The affinity of Syt1 for calcium is indeed strongly enhanced by the presence of the anionic lipids phosphatidylserine (PS) and phosphatidylinositol (4,5)-biphosphate (PIP2)^13,14^. Ca^2+^ binding effectively neutralizes electrostatic repulsion between the target membrane and the Ca^2+^-binding pocket, allowing non-polar residues located at the tip of each calcium loops to insert into the membrane^15–18^ (Fig. 1A).

In addition, a stretch of four consecutive lysine residues (K324–K327) also referred to as a “polylysine patch”, located at the side of Syt1 C2B domain, has been found to bind to PIP2 lipids clustered on the plasma membrane in the absence of Ca^2+^^10,13,19–22^ (Fig. 1A). This interaction is likely important for the initial stage of docking of the synaptic vesicles to the plasma membrane.

Different partially overlapping hypothesis have been advanced to explain how Syt1 couples Ca^2+^ binding to membrane fusion. Syt1 could act as a clamp to prevent SNARE-driven spontaneous fusion, possibly by forming oligomers on membranes, which is removed upon Ca^2+^ binding^23–26^. Syt1 could also simply accelerate fusion by reducing the gap between the synaptic vesicle and the plasma membrane, and/or destabilize the membrane by performing bending work in reaction to binding Ca^2+^, helping reducing the high energy barrier associated with membrane fusion^27–30^. In all cases, the core of Syt1 action is its Ca^2+^-dependent membrane binding and insertion that serves as a power stroke to activate fusion. The precise biochemical and biophysical mechanisms however remain to be understood. One open question is the specific roles of C2A and C2B domains. Mutating the C2A Ca^2+^-binding site leads to a significant decrease in evoked neurotransmitter release in vivo, while a similar mutation in the C2B Ca^2+^-binding site completely abolishes evoked release in vivo^31,32^. Various experimental approaches have attempted to quantify the relative contribution of C2A and C2B in Syt1-membrane interactions in vitro: liposome sedimentation assays^33–35^, stopped-flow fluorescence resonance energy transfer (FRET)^16,36,37^, microscale thermophoresis (MST)^13^, single-molecule force spectroscopy with AFM^38^ and optical tweezers^39^. They generated different results even using similar constructs that likely originate from the technical differences between assays (soluble Syt1 diffusing freely in solution or immobilized on a surface, interacting with liposomes or supported bilayers). Hence the experimental conditions are critical and should reflect the situation in vivo as closely as possible.

We provided a new approach to probe Syt1 interactions with membranes by the use of a Surface Force Apparatus (SFA), that allows for the measurement of the free energy of interaction between two opposite surfaces as a function of their separation distance. We functionalized both surfaces with lipid bilayers and anchored the cytosolic portion of Syt1 to one of the bilayers in a physiologically relevant surface concentration, as shown schematically in Fig. 1B. As such this set up is to our knowledge the closest in vitro simulation of a synaptic vesicle approaching the plasma membrane. We previously showed that the presence of divalent ions allowed for Syt1 to rearrange its C2 domains in an optimal configuration during confinement between membranes, reaching a maximal binding energy of 18 k_B_T per protein in Ca^2+^^40^. However we could not distinguish if both domains contributed equally to this interaction, or if one was predominant. For that we conduct here a mutagenesis study of Syt1 interactions with anionic membranes. By neutralizing Ca^2+^-binding sites of either C2A, C2B, or both domains, we probe the specific roles of each domains in Syt1 membrane binding energetics and configuration.

## Materials and methods

### DNA constructs

The wildtype (WT) Syt1 DNA construct used in this study was generated by cloning the entire cytoplasmic domain (residues 83 to 421) of rat synaptotagmin-1 into pGEX6p-1 (GE Healthcare, Marlborough, MA) using restriction sites XhoI and NotI. A 12 × histidine residue tag was added upstream (N-terminal of the protein) using BamHI and XhoI. Two residues, C277A and E269C, were mutated to allow for subsequent fluorescent labeling. To this WT Syt1 background, additional sets of mutations were created: polylysine patch mutation in C2B (referred to as KAKA: K326A, K327A), Ca^2+^-binding mutation in C2A (referred to as C2aB: D178A, D230A, D232A), polylysine patch and Ca^2+^-binding mutation in C2B (referred to as C2Ab: K326A, K327A, D309A, D363A, D365A) and polylysine patch and Ca^2+^-binding mutation of both C2A and C2B (referred to as C2ab: D178A, D230A, D232A, K326A, K327A, D309A, D363A, D365A). All mutations were introduced using a QuikChange mutagenesis kit (Agilent Technologies, Santa Clara, CA).

### Proteins expression and purification

All constructs were transformed and grown in Escherichia coli BL21(DE3) to an OD 600 ~ 0.8 and the expression was induced with 0.5 mM isopropyl b-D-1-thiogalactopyranoside (IPTG). The cells were harvested after 4 h at 37 °C and suspended in lysis buffer (25 mM HEPES, pH 7.4, 500 mM KCl, 1 mM MgCl_2_, 1 mM CaCl_2_, 15 mM Imidazole, 0.4 mM TCEP, 10% glycerol, 1% Triton X-100, protease inhibitors). The samples were lysed using a cell disrupter, and the lysates were supplemented with 0.1% polyethylimine before centrifugation (35,000 rpm for 30 min). The supernatants were loaded onto Ni–NTA (Qiagen, Valencia, CA) beads (3–4 h or overnight at 4 °C) with 10 μL of Benzonase (2000 units). The beads were washed with 20 mL of lysis buffer with 0.1% Triton X-100, then re-suspended in 5 mL of lysis buffer supplemented with 10 μg/mL of DNAse I, 10 μg/mL of RNAseA and 10 μL of Benzonase, and incubated at room temperature for 1 h. Subsequently, the beads were rinsed quickly with 10 mL of high salt buffer (25 mM HEPES, pH 7.4, 1 M KCl, 1 mM MgCl_2_, 1 mM CaCl_2_, 15 mM Imidazole, 0.4 mM TCEP, 10% glycerol) to remove nucleotide contamination, and washed several times with 25 mM HEPES, pH 7.4, 500 mM KCl, 50 mM Imidazole, 1 mM MgCl_2_, 1 mM CaCl_2_, 0.4 mM TCEP, 10% glycerol. Proteins were eluted off the nickel beads in 25 mM HEPES, pH 7.4, 400 mM KCl, 500 mM Imidazole, 0.5 mM CaCl_2_, 0.4 mM TCEP, 10% glycerol. The GST tag was cleaved overnight at 4 °C using Prescission protease, and then removed with a 1 h room temperature incubation in Glutathione-Sepharose (Thermo Fisher Scientific, Grant Island, NY). The proteins were then run on a size exclusion chromatography column (Superdex 75 16/60 High load) equilibrated with 25 mM HEPES, pH 7.4, 150 mM KCl, 0.4 mM TCEP and further purified by cation-exchange chromatography (MonoS). All chromatography was carried out with AKTA (GE Healthcare, Marlborough, MA). The protein concentration was determined with a Bradford assay using BSA as a standard. The 260 nm/280 nm ratios were measured to check nucleotide contamination. Proteins were flash frozen and stored at − 80 °C with 20% glycerol.

### Surface forces measurements

The force-distance measurements were done with a home-built SFA similar to the original Israelachvili design^41^. Briefly, back-silvered mica surfaces were glued on cylindrical glass disks (R ~ 2 cm) with UV-cured glue (NOA81, Norland Optics), then a monolayer of 1,2-dimyristoyl-sn-glycero-3-phosphoethanolamine (DMPE) was deposited on both surfaces at an area/molecule of 0.4 nm^2^ using a home-built Langmuir–Blodgett trough^42^. DMPE binds strongly to mica, creating a stable inner monolayer on both surfaces. Next, on one surface we deposited an outer layer of 95% 1-palmitoyl-2-oleoyl-sn-glycero-3-phosphocholine (POPC) and 5% 1,2-dioleoyl-sn-glycero-3-[(N-(5-amino-1carboxypentyl)iminodiacetic acid)succinyl] nickel (DGS-NTA(Ni)) with an area/molecule of 0.4 nm^2^, and on the other surface an outer layer of 80% POPC, 15% 1,2-dioleoyl-sn-glycero-3-phospho-L-serine (DOPS), and 5% L-α-phosphatidylinositol-4,5-bisphosphate (PIP2) was deposited at an area/molecule of 0.5 nm^2^. The POPC/DOPS/PIP2 membrane was kept immersed in 25 mM HEPES, 150 mM KCl, with 0.5 mM of EGTA, and in certain cases, 0.5 mM of free Ca^2+^ buffer (calculated using Maxchelator, maxchelator.stanford.edu). The 95% POPC, 5% DGS-NTA(Ni) membrane was immersed in a small vial of the same buffer (~ 3 mL volume) into which ~ 5 μL of ~ 2 mg/mL 12×His-Syt1 was injected and mixed well via pipet. After 1 h of protein immersion, the small vial was transferred twice into clean buffer solution (~ 200 mL volume) to remove unbound protein. Finally both surfaces were carefully transferred under buffer into the SFA chamber. One surface was mounted on a spring with the other on a stiff mount in a crossed-cylinder geometry. The distance was measured via multiple beam interferometry and the force by spring deflection. For each condition we measured at least 2 independent experimental setups, with at least 6 independent contact locations to demonstrate reproducibility. Error bars represent standard errors over the independent contact locations. Statistical significance was assessed by a two-tailed unpaired Student’s T test.

## Results

We used a surface force apparatus (SFA) to directly measure the interaction energy between a lipid membrane decorated with a cytoplasmic portion of Syt1 and an anionic membrane composed of PC/PS/PIP2 lipids. A typical SFA measurement consists of several cycles of approach and separation of the two surfaces. The distance is measured interferometrically (~ 1 Å resolution) simultaneously to the corresponding force with a cantilever spring (force resolution ~ 100 nN) every ~ 10 s. Given the specific geometry of the SFA, this force F is directly linked to the surface energy per unit area W by the Derjaguin’s approximation:

$$\mathrm{W}=\frac{\mathrm{F}}{2\mathrm{\pi R}}$$

where R is the radius of the surfaces.

Between the end of approach and the beginning of separation, a 1 h contact time (t_c_ = 1 h) is applied in the standard procedure. The surfaces were initially immersed in 25 mM HEPES, 150 mM KCl buffer with either 0.5 mM EGTA or 0.5 mM free Ca^2+^. As described previously^40^, repulsive forces were measured during Syt1 approach to the opposite bilayer starting at the distance D ~ 25–30 nm that were attributed to a steric interaction between Syt1 chains and the membrane. The resulting exponential repulsion was well fitted by a mushroom polymer model from which the surface density of the protein Γ could be extracted. One can then easily find the free interaction energy per molecule of Syt1 by the relation:

$${\mathrm{E}}_{\mathrm{Syt}1}=\frac{\mathrm{W}}{\Gamma }$$

**Figure 2:**
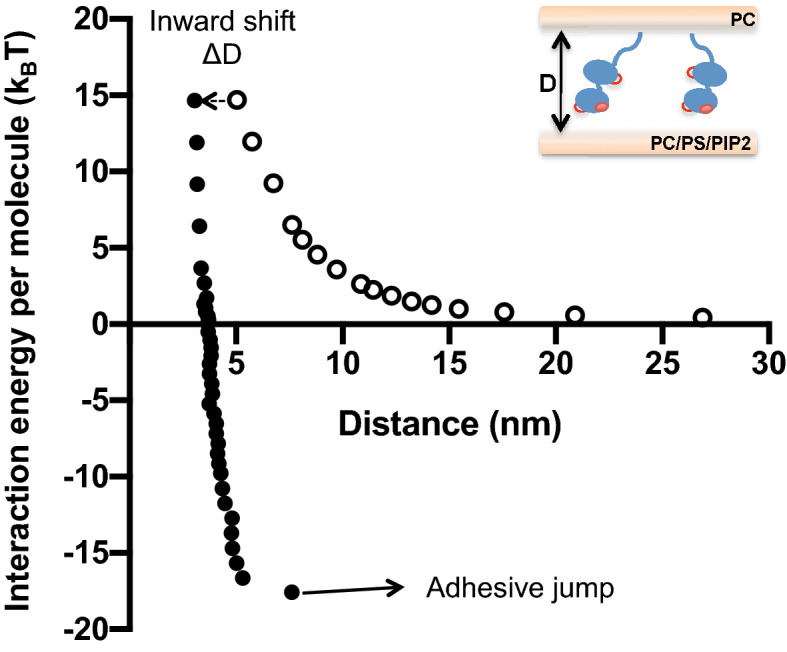
Representative Energy vs Distance curve of a typical SFA experiment of a Syt1-coated PC membrane opposing a 80%PC/5%PIP2/15%PS membrane in presence of calcium. Empty symbols are during approach while filled symbols are during separation.

As such we measure the energy per molecule as a function of the distance between the membranes, allowing for distance-dependent probing of the energetics of confinement and binding of different versions of Syt1, in several buffer conditions. The lipid composition of the membranes was kept identical in all measurements (95%PC/5%NTA(Ni) in the Syt1 *cis*-bilayer and 85%PC/15%PS/5%PIP2 in the target bilayer). A representative SFA run of wild type Syt1 (WT) in Ca^2+^ is shown in Fig. 2. For each run we measured the distance shift ΔD occurring during the contact time t_c_ that corresponds to the variation of the distance between the last point of approach and the first point of separation (Fig. 2, dotted arrow). At the end of separation, in case of adhesion, the surfaces suddenly jump out of contact (indicated by the “adhesive jump” arrow in Fig. 2). The corresponding adhesion energy is the membrane binding energy of Syt1.

We already showed that divalent ions strongly enhanced the membrane binding energy of Syt1, going from ~ 6 k_B_T in EGTA, to ~ 10 k_B_T in Mg^2+^ and reached a maximum of ~ 18 k_B_T in Ca^2+^. The increase in membrane binding energy was correlated with a molecular rearrangement of Syt1 during confinement as evidenced by a corresponding reduction of the interbilayer distance (distance shift ΔD)^40^. Here we expand on this analysis by introducing mutations to specific sites of the protein that impair either its Ca^2+^-independent or Ca^2+^-dependent binding (Fig. 1A).

**Figure 3:**
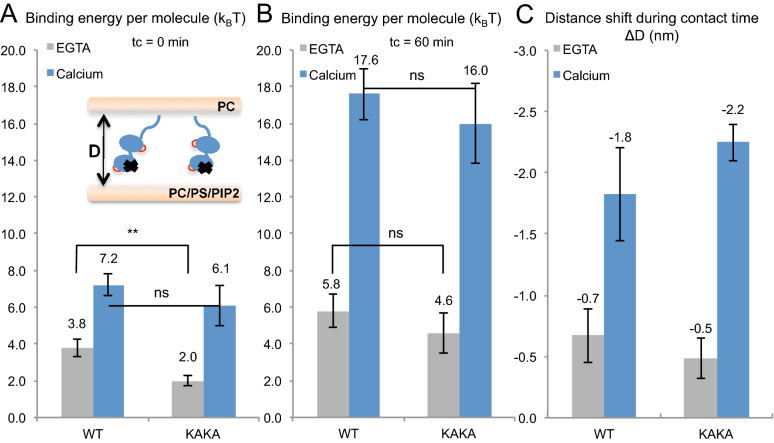
Averaged binding energies of wild type Syt1 (WT) and KAKA mutant to 80%PC/5%PIP2/15%PS membrane in EGTA (grey) and calcium (blue). (**A**) Binding energy per molecule of Syt1 when no contact time is applied between the approach and the separation of membranes. (**B**) Binding energy per molecule of Syt1 when a 60 min contact time is applied between the approach and the separation of membranes. (**C**) Measured distance shift ΔD during the 60 min contact time between approach and separation. **P < 0.01; ns, not significant.

We first studied the impact of the mutation of the C2B polylysine site (KAKA mutant) on the binding energetics of Syt1. Neutralization of two of the four lysines of the polybasic patch was shown to be sufficient to impair Syt1 binding to PIP2 containing liposomes in the absence of Ca^2+^^21,43^, the so-called KAKA mutation (K326/327A). We compared values obtained when no time was left for equilibration between the end of approach and the beginning of separation (t_c_ = 0, Fig. 3A) to when the surfaces were left in contact for 60 min (t_c_ = 60, Fig. 3B). Similar to the WT, the ion composition of the buffer strongly impacted the binding energy of KAKA to anionic membranes, going from ~ 5 k_B_T in EGTA to ~ 16 k_B_T in Ca^2+^*.* This trend was also observed at short contact times but was less pronounced. Hence the polylysine patch mutation does not impair the protein’s ability to bind Ca^2+^ and to insert its loops in the membrane. This is supported by the inward distance shifts observed during the waiting time (Fig. 3C): the surfaces in contact came closer by ~ 2.2 nm in Ca^2+^. These values are again very similar to the distance shifts measured with the WT, confirming that the C2B polylysine patch of Syt1 does not impact its Ca^2+^-dependent molecular rearrangement. At short contact times however the KAKA mutation reduced the binding energy of WT Syt1 in EGTA by ~ 50%. This is consistent with the proposed role of the polylysine patch as being the primary interacting site of Syt1 with the opposing membrane in the absence of Ca^2+^, docking the protein on the plasma membrane through its interaction with PIP2 lipids^44^. Disruption of this interaction would result in a more random orientation of Syt1 upon the approach of the anionic membrane, leading to the observed decrease in the short time binding energy and reflecting less specific Syt1-membrane interactions. In Ca^2+^ however, the similar binding energies measured with the WT and KAKA mutant show that the Ca^2+^-dependent membrane binding dominates over the polylysine patch interaction with PIP2 even at short contact times. Hence the polylysine patch role would be primarily to initiate and orientate the pre-calcium membrane binding of Syt1 rather than being a main energetic contributor to Syt1 overall membrane binding.

**Figure 4:**
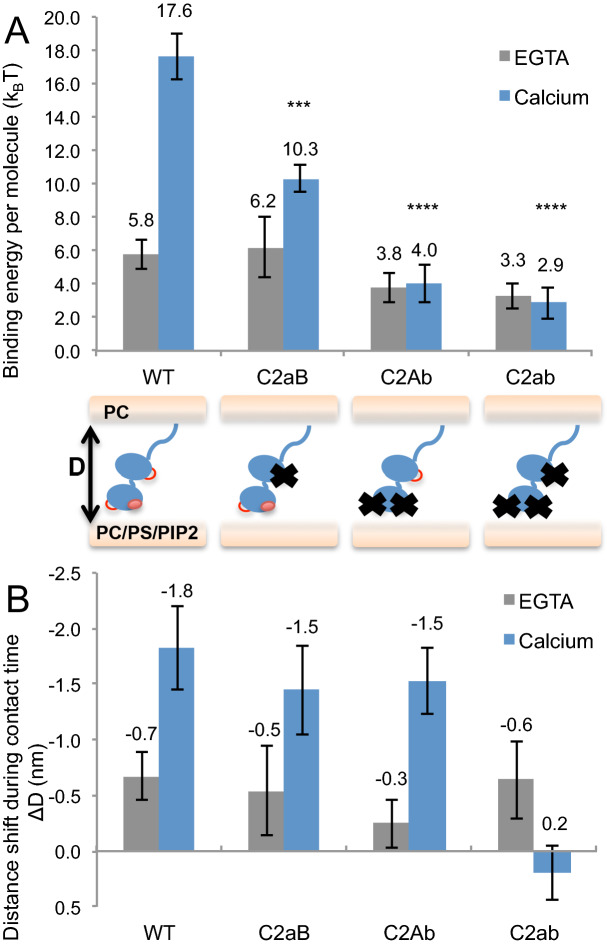
Impact of the disruption of one or both calcium binding sites on the averaged membrane binding energy and interbilayer molecular organization of Syt1. (**A**) Binding energy per molecule of wild type Syt1 (WT), C2A calcium binding mutant (C2aB), C2B calcium binding mutant (C2Ab), and both C2A and C2B calcium binding mutant (C2ab) measured in EGTA (grey) and calcium (blue). (**B**) Corresponding distance shift ΔD measured during the 60 min contact time between approach and separation. ***P < 0.001; ****P < 0.0001 as compared to WT.

We then investigated the impact of the neutralization of the Ca^2+^-binding site of C2A (C2aB), C2B (C2Ab), or both domains (C2ab), on the membrane binding energy of Syt1 in Ca^2+^ and on the molecular rearrangement. C2Ab and C2ab also include the polylysine patch mutation. Results for the long contact time where the maximum binding energy was reached are shown in Fig. 4. As expected, neutralization of the Ca^2+^-binding sites did not have any significant impact on the binding energies in EGTA. The difference between the mutants was more apparent in the presence of Ca^2+^. Strikingly, the Ca^2+^-dependent binding energy increase observed in the WT and KAKA mutant was completely abolished in C2Ab (i.e., by neutralizing the C2B). In fact, the binding energies in Ca^2+^ were similar to the ones in EGTA for both C2Ab and C2ab mutants. Hence we conclude that Ca^2+^ binding to the C2B domain is a necessary condition for the increase of binding energy of Syt1 to the membrane. A functional C2A domain is not sufficient to completely recover the C2B Ca^2+^-binding impairment. However in looking at the distance shifts, differences arise between the C2Ab and the C2ab mutants: during confinement between the surfaces, the C2Ab mutant showed a similar molecular rearrangement as the WT (~ 1.5 nm), whereas no sign of molecular rearrangement was measured with the C2ab mutant. This confirms that the Syt1 rearrangement between membranes originates from the Ca^2+^-dependent interactions of Syt1. Interestingly, Ca^2+^ binding to the C2A domain alone can drive this rearrangement, without leading to an increase in binding energy.

The finding that mutating the C2B Ca^2+^-binding site results in a nearly complete loss of the Ca^2+^-dependent binding energy could suggest that C2B is solely responsible for this interaction. In that case, mutating the Ca^2+^-binding site of the C2A domain (C2aB) should not have a noticeable impact on the binding energies. Another possibility could be a “both or none” mode of insertion of C2A and C2B, where the insertion (or non insertion) of one domain would automatically promote (or respectively cancel) the insertion of the other domain. In this hypothesis, mutating the C2A Ca^2+^-binding site (C2aB) would give a similar decrease in the binding energies in Ca^2+^ to that observed when the C2B Ca^2+^-binding site is mutated (C2Ab). However, neither of these scenarios was observed: the binding energy of the C2aB mutant in Ca^2+^ (~ 10 k_B_T) was between that of the WT (~ 18 k_B_T) and the C2Ab mutant (~ 4 k_B_T). Hence the effect of C2A Ca^2+^-binding site mutation is meaningful, yet not as significant as that of the C2B domain. Again, we observed an inward shift of the surfaces during the contact time (ΔD ~ 1.7 nm) similar to that of the WT and the C2Ab mutant*.* The C2B domain likely drives this molecular rearrangement. Hence, having only one of the Ca^2+^-binding sites active is a necessary and sufficient condition to promote a change of Syt1 configuration during confinement. It is also worth noting that the energy measured with the double mutant C2ab in presence of Ca^2+^ likely reflects non-specific membrane binding properties of Syt1, *i.e.* that do not depend on Syt1 Ca^2+^-binding sites, and could originate from other charged residues present in the C2 domains or in the N-terminal linker domain of Syt1.

## Discussion

We measured the impact on the binding energy of Syt1 to anionic membranes of the mutation of specific residues affecting either its Ca^2+^-independent membrane binding properties (via the KAKA mutation) or its Ca^2+^-dependent membrane binding properties (via the neutralization of the Ca^2+^-binding sites of either one or both of its C2 domains). In parallel, the measurement of the evolution of the membrane separation distance during the contact time ΔD allows us to correlate these energetic data with the molecular organization of Syt1 during confinement between the two membranes. While the composition of the lipid bilayers was kept constant in all experiments, we cannot exclude the possibility that Syt1 induces lipid exchange between the outer leaflets. However such exchange would mainly involve POPC and would therefore have very little impact on the overall membrane composition and binding of Syt1. We chose not to include negatively charged lipids in the Syt1-coated bilayer in order to avoid *cis* interactions between Syt1 and its own membrane. The Syt1 interaction with *cis* PS lipids has been found to be screened by ATP under physiological conditions^18^. Hence, our results should capture the primary physiologically-relevant *trans* Syt1 interactions.

Taken together our data provide a mapping of the membrane binding energetics of Syt1 (Table 1). We can first isolate the contribution of the Ca^2+^-binding modules of Syt1 from the total binding energy of Syt1 in Ca^2+^ (E_WT_) by subtracting from the WT the binding energy of the mutant where both C2A and C2B Ca^2+^-binding sites were neutralized (C2ab mutant). The latter is a good estimation of the binding energy of Syt1 that is not specific to its Ca^2+^-binding domains (E_unspecific_, ~ 3 k_B_T), being also the only mutant for which no significant change in the interbilayer distance in Ca^2+^ was measured. We find that ~ 14 k_B_T can be attributed solely to the C2A and C2B membrane binding in Ca^2+^ (E_calcium-specific_).

**Table 1:** Estimations of site-specific membrane binding energy of Syt1 in Ca^2+^ by correlating molecular rearrangements and binding energies of Syt1 mutants.

Total binding energy	E_WT_ = 17.6 ± 1.4 k_B_T
**Estimation of the binding energy distribution**	
Unspecific energy (E_unspecific_)	E_C2ab_ = 2.9 ± 0.9 k_B_T
**Site-specific energy**	
Calcium binding energy (E_calcium-specific_)	E_WT_–E_C2ab_ or E_KAKA_–E_C2ab_ = **14.7 ± 0.6 k**_**B**_**T**
C2A energetic contribution (E_C2A_)	E_C2Ab_–E_C2ab_ = **1.2 ± 0.5 k**_**B**_**T**
C2B energetic contribution (E_C2B_)	E_C2aB_–E_C2ab_ = **7.5 ± 0.5 k**_**B**_**T**
C2A–C2B cooperative gain	E_calcium-specific_–E_C2A_–E_C2B_ = **6.1 ± 0.1 k**_**B**_**T**

We can apply the same logic to quantify the contribution of each C2 domains taken independently. We estimate the contribution of the C2B domain to be ~ 7 k_B_T and that of C2A domain to be ~ 1 k_B_T. If the C2A domain therefore appears energetically negligible, it is still able to bring the membranes closer in Ca^2+^ even in the absence of a functional C2B, as shown by the distance shift measured with the C2Ab mutant (and not observed with the C2ab mutant). The capacity of C2A to drive Syt1 molecular rearrangement independently of the presence of an active C2B rules out the ‘both or none’ hypothesis. Overall, summing up the C2A and C2B energies gives only ~ 8 k_B_T, which does not account for the total ~ 14 k_B_T of Syt1 calcium-specific binding energy estimated earlier. Where does this additional energy come from? How can we explain the fact that the global binding energy of the C2 domains taken together is greater than the sum of its components?

**Figure 5:**
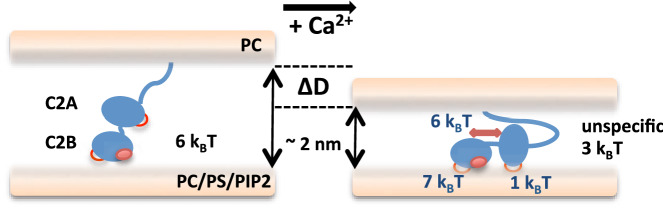
Model for the repartition of the binding energy of Syt1 to anionic membranes between its individual C2A and C2B domains. In the absence of calcium (left), the global binding energy of Syt1 (~ 6 k_B_T) most likely arises from columbic interactions between charged residues and anionic lipids including the polylysine patch. Upon calcium binding (right), C2 domains of Syt1 reorients to bind the membrane in an optimal configuration, leading to an increased binding energy of ~ 18 k_B_T correlated with an interbilayer distance reduction of ~ 2 nm. C2B provides most of the calcium-dependent binding energy (~ 7 k_B_T), while C2A brings a negligible binding energy (~ 1 k_B_T) but acts as a facilitator of C2B binding, adding another ~ 6 k_B_T of cooperative binding when both C2A and C2B calcium binding sites are active. Residual energy (~ 3 k_B_T) comes from unspecific binding sites of Syt1.

We thus propose that C2A and C2B act cooperatively, with both domains required to maximize the binding energy (Fig. 5). Such an arrangement could explain both the energies and distance shifts observed. According to this model, if one of the C2 domains is neutralized, either C2A or C2B, the resulting binding energy will be decreased by more than the intrinsic binding energy of the individual mutated domain. On the opposite, if both domains are active, the total binding energy will be higher than the addition of the intrinsic binding energies of both individual domains. The ~ 7 k_B_T energy drop measured when mutating only C2A accounts for both the loss of cooperativity and the intrinsic binding energy of C2A. Similarly, the ~ 14 k_B_T energy loss measured when mutating only C2B also reflects the loss of cooperativity and the intrinsic binding energy of C2B. This gives an energetic map were C2B provides most of the Ca^2+^-binding energy of Syt1 (~ 7 k_B_T) while C2A only accounts for a negligible energy (~ 1 k_B_T) but brings an extra ~ 6 k_B_T when both domains are active and cooperate to maximize Syt1 membrane binding in Ca^2+^. We estimate the coupling energy arising from the cooperativity between both domains to be in the same range as the intrinsic binding energy of C2B. As such the presence of an active tandem C2A domain would double the apparent binding energy of C2B. The presence of both functional domains however does not seem to be mandatory to promote a reorientation of Syt1 during confinement, as the presence of at least one active C2 domain is enough for Syt1 to reduce the distance between membranes by a couple of nanometers.

The attribution of 7 k_B_T to the C2B intrinsic binding energy is coherent with a recent single molecule optical tweezers study that measured a binding energy of isolated C2B to PS/PIP2 membranes of ~ 7 k_B_T. They were not able to measure a binding energy for the isolated C2A, likely because the intrinsic contribution of C2A is too low to be detected, in line with our estimation of ~ 1 k_B_T^39^.

A cooperative behavior of tandem C2A and C2B domains was suspected by previous studies. EPR analysis of soluble C2A-C2B binding to anionic membranes showed that the extent of membrane penetration of tandem C2A and C2B domains (C2AB) was deeper than that of isolated C2A and C2B by about 6–7 Å^15^. Recent stopped-flow kinetics measurements showed that the dissociation from anionic liposomes of soluble Syt1 tandem C2 domains was significantly slower than both of its individual domains, another sign of cooperativity between C2A and C2B in Ca^2+^-membrane binding^37^. Cooperative behavior of C2A and C2B domains has also been hinted at SNARE binding studies, wherein C2AB exhibits higher affinity and efficacy, than individual C2 domains^32,45^. Overall, our data provide an energetic perspective on the previous observations of cooperative behavior of the C2 domains of Syt1, which may explain the presence of multiple C2 domains in Synaptotagmins and other proteins with C2 domains^46^. The molecular origin of this apparent cooperativity remains to be elucidated.

The predominance of C2B function over C2A is also observed in vivo. The neutralization of the C2B Ca^2+^-binding site decreased by more than 95% the evoked neurotransmitter release in Drosophila^31^ and inhibited synchronous transmitter release in cultured hippocampal neurons^47^. Similarly, the mutation of a single hydrophobic residue of the Ca^2+^-binding pocket of C2B required for its Ca^2+^-dependent membrane penetration disrupted evoked transmitter release^32^. Hence the Ca^2+^-binding site of C2B and the membrane insertion of its loops are essential for synaptic transmission. However, a similar mutation in the C2A domain only resulted in a 50% decrease in evoked transmitter release^32^, in line with a facilitatory role of C2A. Its ability to bind Ca^2+^ is not required for the Ca^2+^-dependent properties of transmission^48^. So far there is no definite explanation to why C2B is physiologically dominant. Our data accommodate well with the in vivo studies, showing from an energetic perspective a predominant role of C2B, and an auxiliary, facilitatory role of C2A in Syt1 membrane binding.

C2B has been found to be functionally critical in other types of interactions. For example, soluble C2AB domain induced curvature on anionic liposomes in Ca^2+^, a process driven by C2B^28,29^. Moreover, it was proposed that Syt1 could oligomerize on PIP2-containing membrane in the absence of Ca^2+^, possibly preventing SNAREs from full zippering; Syt1 oligomers then disassemble upon Ca^2+^ binding^23–26,49–51^. Interestingly, the disruption of the oligomer depended on the ability of C2B, and not C2A, to bind Ca^2+^^24^. The stronger membrane binding energy measured here for C2B relative to C2A might explain why C2B is the main functional unit of Syt1. Further knowledge of the precise configuration and interacting partners of Syt1, that are still the subject of intense research^52–54^, is needed to better understand how exactly these energies are translated into the overall fusion process.

## Conclusion

Understanding the site-specific membrane binding properties of Syt1 is an important step towards a clear picture of its fundamental role in neurotransmission. Here we show that Syt1 C2B domain energetically predominates Ca^2+^-dependent membrane binding, while C2A domain seems to have an important role as a facilitator of C2B binding. Both domains cooperate to position Syt1 in the most favorable configuration for a maximized binding energy. Our results are in agreement with the physiological roles of C2A and C2B in mediating evoked neurotransmitter release.

## References

[1] Brose N, Petrenko A, Sudhof T, Jahn R (1992). Synaptotagmin: A calcium sensor on the synaptic vesicle surface. Science.

[2] Südhof TC, Rothman JE (2009). Membrane fusion: Grappling with SNARE and SM proteins. Science.

[3] Chapman ER (2008). How does synaptotagmin trigger neurotransmitter release?. Annu. Rev. Biochem..

[4] Fernández-Chacón R (2002). Synaptotagmin I functions as a calcium regulator of release probability. Nature.

[5] Sutton RB, Davletov B, Berghuis A, Sudhof T, Sprang SR (1995). Structure of the first C2 domain of synaptotagmin I: a novel Ca^2+^/phospholipid-binding fold. Cell.

[6] Fernandez R (2001). Three-dimensional structure of the synapotagmin 1 C2B-domain: Synaptotagmin 1 as a phospholipid binding machine. Neuron.

[7] Fernandez R (2002). Structure/function analysis of Ca2 binding to the C2A domain of synaptotagmin 1. J. Neuro Sci..

[8] Ubach J, Zhang X, Shao X, Südhof TC, Rizo J (1998). Ca^2+^ binding to synaptotagmin: How many Ca^2+^ ions bind to the tip of a C2-domain?. J. Neurosci..

[9] Südhof TC (2004). The synaptic vesicle cycle. Annu. Rev. Neurosci..

[10] Bradberry MM, Bao H, Lou X, Chapman ER (2019). PIP _2_ drives Ca^2+^-independent membrane penetration by the tandem C2 domain proteins synaptotagmin-1 and Doc2β. J. Biol. Chem..

[11] Striegel AR (2012). Calcium binding by synaptotagmin’s C2A domain is an essential element of the electrostatic switch that triggers synchronous synaptic transmission. J. Neurosci..

[12] Rizo J, Chen X, Araç D (2006). Unraveling the mechanisms of synaptotagmin and SNARE function in neurotransmitter release. Trends Cell Biol..

[13] Den Van Bogaart G, Meyenberg K, Diederichsen U, Jahn R (2012). Phosphatidylinositol 4,5-bisphosphate increases Ca^2+^ affinity of synaptotagmin-1 by 40-fold. J. Biol. Chem..

[14] Schiavo G, Gu QM, Prestwich GD, Söllner TH, Rothman JE (1996). Calcium-dependent switching of the specificity of phosphoinositide binding to synaptotagmin. Proc. Natl. Acad. Sci..

[15] Herrick DZ, Sterbling S, Rasch KA, Hinderliter A, Cafiso DS (2006). Position of synaptotagmin I at the membrane interface: Cooperative interactions of tandem C2 domains. Biochemistry.

[16] Hui E, Bai J, Chapman ER (2006). Ca^2+^-triggered simultaneous membrane penetration of the tandem C2-domains of synaptotagmin I. Biophys. J..

[17] Chapman ER, Davis AF (1998). Direct interaction of a Ca^2+^-binding loop of synaptotagmin with lipid bilayers. J. Biol. Chem..

[18] Park Y (2012). Controlling synaptotagmin activity by electrostatic screening. Nat. Struct. Mol. Biol..

[19] Loewen CA, Lee S, Shin YK, Reist NE (2006). C2B polylysine motif of synaptotagmin facilitates a Ca^2+^-independent stage of synaptic vesicle priming in vivo. Mol. Biol. Cell.

[20] Park Y (2015). Synaptotagmin-1 binds to PIP2-containing membrane but not to SNAREs at physiological ionic strength. Nat. Struct. Mol. Biol..

[21] Bai J, Tucker WC, Chapman ER (2004). PIP2 increases the speed of response of synaptotagmin and steers its membrane-penetration activity toward the plasma membrane. Nat. Struct. Mol. Biol..

[22] Lai Y, Lou X, Diao J, Shin Y-K (2015). Molecular origins of synaptotagmin 1 activities on vesicle docking and fusion pore opening. Sci. Rep..

[23] Wang J (2014). Calcium sensitive ring-like oligomers formed by synaptotagmin. Proc. Natl. Acad. Sci..

[24] Zanetti MN (2016). Ring-like oligomers of synaptotagmins and related C2 domain proteins. Elife.

[25] Wang J (2017). Circular oligomerization is an intrinsic property of synaptotagmin. Elife.

[26] Rothman JE, Krishnakumar SS, Grushin K, Pincet F (2017). Hypothesis—buttressed rings assemble, clamp, and release SNAREpins for synaptic transmission. FEBS Lett..

[27] Wu Z, Schulten K (2014). Synaptotagmin’s role in neurotransmitter release likely involves Ca^2+^-induced conformational transition. Biophys. J..

[28] Hui E, Johnson CP, Yao J, Dunning FM, Chapman ER (2009). Synaptotagmin-mediated bending of the target membrane is a critical step in Ca^2+^-regulated fusion. Cell.

[29] Martens S, Kozlov M, McMahon HT (2007). How synaptotagmin promotes membrane fusion. Science.

[30] François-Martin C, Rothman JE, Pincet F (2017). Low energy cost for optimal speed and control of membrane fusion. Proc. Natl. Acad. Sci..

[31] Mackler JM, Drummond JA, Loewen CA, Robinson IM, Reist NE (2002). The C2B Ca^2+^-binding motif of synaptotagmin is required for synaptic transmission in vivo. Nature.

[32] Paddock BE (2011). Membrane penetration by synaptotagmin is required for coupling calcium binding to vesicle fusion in vivo. J. Neurosci..

[33] Wang S, Li Y, Ma C (2016). Synaptotagmin-1 C2B domain interacts simultaneously with SNAREs and membranes to promote membrane fusion. Elife.

[34] Kuo W, Herrick DZ, Ellena JF, Cafiso DS (2009). The calcium-dependent and calcium-independent membrane binding of synaptotagmin 1: two modes of C2B binding. J. Mol. Biol..

[35] Kuo W, Herrick DZ, Cafiso DS (2011). Phosphatidylinositol 4,5-bisphosphate alters Synaptotagmin 1 membrane docking and drives opposing bilayers closer together. Biochemistry.

[36] Pérez-Lara Á (2016). PtdInsP2 and PtdSer cooperate to trap synaptotagmin-1 to the plasma membrane in the presence of calcium. Elife.

[37] Tran HT, Anderson LH, Knight JD (2019). Membrane binding cooperativity and co-insertion by C2AB tandem domains of synaptotagmins 1 and 7. Biophys. J..

[38] Takahashi H, Shahin V, Henderson RM, Takeyasu K, Edwardson JM (2010). Interaction of synaptotagmin with lipid bilayers, analyzed by single-molecule force spectroscopy. Biophys. J..

[39] Ma L (2017). Single-molecule force spectroscopy of protein-membrane interactions. Elife.

[40] Gruget C (2018). Rearrangements under confinement lead to increased binding energy of Synaptotagmin-1 with anionic membranes in Mg^2+^ and Ca^2+^. FEBS Lett..

[41] Israelachvili JN, Adams GE (1978). Measurement of forces between two mica surfaces in aqueous electrolyte solutions in the range 0–100 nm. J. Chem. Soc. Faraday Trans.

[42] Perez E, Wolfe J (1994). A simple, cheap, clean, reliable, linear, sensitive low-drift transducer for surface pressure. Langmuir.

[43] Li L (2006). Phosphatidylinositol phosphates as co-activators of Ca^2+^ binding to C2 domains of synaptotagmin 1. J. Biol. Chem..

[44] Lin C (2014). Control of membrane gaps by synaptotagmin-Ca^2+^ measured with a novel membrane distance ruler. Nat. Commun..

[45] Tucker WC, Weber T, Chapman ER (2004). Reconstitution of Ca^2+^-regulated membrane fusion by synaptotagmin and SNAREs. Science.

[46] Volynski KE, Krishnakumar SS (2018). ScienceDirect Synergistic control of neurotransmitter release by different members of the synaptotagmin family. Curr. Opin. Neurobiol..

[47] Nishiki T-I, Augustine GJ (2004). Dual roles of the C2B domain of synaptotagmin I in synchronizing Ca^2+^-dependent neurotransmitter release. J. Neurosci..

[48] Robinson IM, Ranjan R, Schwarz TL (2002). Synaptotagmins I and IV promote transmitter release independently of Ca^2+^-binding in the C2A domain. Nature.

[49] Bello OD (2018). Synaptotagmin oligomerization is essential for calcium control of regulated exocytosis. PNAS.

[50] Tagliatti E (2019). Synaptotagmin 1 oligomers clamp and regulate different modes of neurotransmitter release. bioRxiv.

[51] Ramakrishnan S (2019). Synaptotagmin oligomers are necessary and can be sufficient to form a Ca^2+^-sensitive fusion clamp. FEBS Lett..

[52] Zhou Q (2015). Architecture of the synaptotagmin-SNARE machinery for neuronal exocytosis. Nature.

[53] Zhou Q (2017). The primed SNARE-complexin-synaptotagmin complex for neuronal exocytosis. Nature.

[54] Grushin K (2019). Structural basis for the clamping and Ca^2+^ activation of SNARE-mediated fusion by synaptotagmin. Nat. Commun..

